# Evaluating the Contribution of Gut Microbiota to the Variation of Porcine Fatness with the Cecum and Fecal Samples

**DOI:** 10.3389/fmicb.2016.02108

**Published:** 2016-12-23

**Authors:** Maozhang He, Shaoming Fang, Xiaochang Huang, Yuanzhang Zhao, Shanlin Ke, Hui Yang, Zhuojun Li, Jun Gao, Congying Chen, Lusheng Huang

**Affiliations:** State Key Laboratory of Pig Genetic Improvement and Production Technology, Jiangxi Agricultural UniversityNanchang, China

**Keywords:** gut microbiome, fatness, swine, two-part model analysis, 16S rRNA gene

## Abstract

Microbial community in gastrointestinal tract participates in the development of the obesity as well as quite a few metabolic diseases in human. However, there are few studies about the relationship between gut microbiota and porcine fatness. Here, we used high-throughput sequencing to perform 16S rRNA gene analysis in 256 cecum luminal samples from *Erhualian* pigs and 244 stools from *Bamaxiang* pigs, and adopted a two-part model statistical method to evaluate the association of gut microbes with porcine fatness. As the results, we identified a total of 6 and 108 operational taxonomic units (OTUs), and 9 and 10 bacterial taxa which showed significant associations with fatness traits in the stool and cecum samples, respectively. Cross-validation analysis indicated that gut microbiome showed the largest effect on abdominal adipose by explaining 2.73% phenotypic variance of abdominal fat weight. Significantly more fatness-associated OTUs were identified in the cecum samples than that in the stools, suggesting that cecum luminal samples were better used for identification of fatness-associated microbes than stools. The fatness-associated OTUs were mainly annotated to *Lachnospiraceae*, *Ruminococcaceae*, *Prevotella*, *Treponema*, and *Bacteroides*. These microbes have been reported to produce short-chain fatty acids by fermenting dietary indigested polysaccharide and pectin. The short-chain fatty acids can regulate host body energy homeostasis, protect host from inflammation and inhibit fat mass development. Our findings suggested that the gut microbiome may be an important factor modulating fatness in pigs.

## Introduction

Obesity has been becoming one of the major health problems for humans, which is characterized by excessive fat accumulation, and imbalanced energy intake and expenditure, and accompanies with low grade of systemic and chronic inflammation. It is associated with a wide range of pathological disturbances in metabolic organs and then predisposes toward type 2 diabetes mellitus (T2DM) (Hanning and Diaz-Sanchez, 2015) and cardiovascular disease (Kahn et al., 2006; Canfora et al., 2015). Further, obesity is also related to certain types of cancer, osteoarthritis, and asthma. In pigs, fatness has been regarded as a typically complex and economic trait in pig production. It brings low feed conversion rate and unfavorable fat mass. Dissection of the mechanism for the fatness in pigs not only benefits the pig industry but also provides important information for understanding human obesity, because pigs possess greater similarity with humans in nutritional and metabolic physiology compared to other animal models (Swindle et al., 2012). Fatness is affected by many factors, such as genetics, nutrition, and lifestyle as well as gut microbiome. More and more studies in humans have shown that obesity is related to the gut microbiota (Ley et al., 2006; Turnbaugh et al., 2009; Munoz-Garach et al., 2016).

Mammalian gastrointestinal tract is resided by a complex, diverse, and dynamic community of symbiotic microbes that continuously interact with the host (Hooper et al., 2002; Backhed et al., 2005). Gut microbiota has demonstrated the great significance to animals by providing a large amount of functions that host lacks, such as fermenting undigested energy substrates, stimulating the host immune system development, participating in the metabolic processes, preventing growth of harmful and pathogenic bacteria and so on (Guarner and Malagelada, 2003). The relationship between human obesity and gut microbiota composition has been established for several decades with the hallmark study by Backhed et al. (2004) which demonstrated that the gut microbiota as an environmental factor modulates fat storage. Since then, the role of the gut microbiota in the pathogenesis of obesity has become a vigorous research area. The further studies showed that obesity is associated with the changes of two dominant phylum-level bacteria of *Bacteroidetes* and *Firmicutes* in the gut (Mariat et al., 2009; Gerritsen et al., 2011). A reduction of bacterial diversity and the altered metabolic pathways were demonstrated to associate obesity from a comparison study in obese and lean twins (Turnbaugh et al., 2009). An endotoxin-producing “obese microbe” was isolated from an obese human and was confirmed to cause the obesity in the germfree mice (Fei and Zhao, 2013). In pigs, the studies reported by Guo et al. (2008a,b), Wall et al. (2009), and Luo et al. (2012) inferred that the gut microbiota might participate in the process of fat storage and should be correlated with the porcine adiposity formation. In addition, Yang et al. (2016) identified tens of fatness-associated bacteria including *Escherichia* spp. which showed a higher relative abundance in high fatness pigs. Overall, accumulating evidences suggest that the endotoxin-induced inflammation, dysbiosis of gut microbiota composition and *Firmicutes/Bacteroidetes* ratio are involved in the development of obesity. But the mechanism remains controversial (Turnbaugh et al., 2006, 2009; Cani et al., 2007; Wen et al., 2008).

*Bamaxiang* is a Chinese indigenous mini pig breed, and *Erhualian* is another Chinese indigenous pig breed which is famous for its high prolificacy. Both breeds show a higher fatness than Western pig breeds (Ai et al., 2013). In this study, we evaluated the association of the gut microbiome with porcine fatness in both faces and cecum luminal samples.

## Materials and Methods

### Animals and Phenotype Measurement

A total of 244 *Bamaxiang* and 256 *Erhualian* pigs were used in this study. The two pig populations were raised in the same farm house. All experimental pigs were fed two times a day using the corn-soybean feed containing 14∼16% of crude protein, 8% of coarse fiber, 3,100 kJ of digestible energy and 0.85% of lysine, and given an *ad libitum* water. All animals were healthy and did not receive any antibiotic treatment within 2 months before slaughter. The experimental pigs were slaughtered at 300 ± 3 days. The fatness traits including backfat thickness (measured at shoulder, chest and waist, and defined as ShoulderBF, ChestBF, WaistBF, and AverageBF) and fat mass (including LeafFatWt and AbdomenFatWt) (**Table 1**) were separately measured by the vernier caliper and electronic platform balance. All animal works were conducted according to the guidelines for the care and use of experimental animals established by the Ministry of Agriculture of China. The project was specially approved by Animal Care and Use Committee (ACUC) in Jiangxi Agricultural University.

**Table 1:** Summary of gut microbial structure identified in the cecum and feces.

Sample	Phylum	Family	Genus	C4pcOperational taxonomic unit (OTU)
Cecum	15 (18^∗^)	45 (73)	45 (99)	524 (2,038)
Feces	16 (17)	43 (55)	42 (57)	610 (1,660)

*^∗^The numbers in brackets were the data before quality control*.

### Fecal and Cecum Luminal Sample Collection and DNA Extraction

The luminal contents of cecum were collected from the *Erhualian* population when pigs were killed in the slaughter house. The fecal samples of the *Bamaxiang* population were harvested from the rectum before the pigs were transported to the slaughter house. All samples were collected in the 7-ml sterilized plastic centrifuge tubes and dipped in liquid nitrogen immediately. After transported to the laboratory, the samples were stored at -80°C freezer until used. DNA was extracted from fecal and luminal samples with QIAamp DNA Stool Mini Kit (Qiagen, Germany) according to the manufacturer’s protocol (McOrist et al., 2002). The concentration and integrity of DNA were measured by the Nanodrop-1000 and the 0.8% agarose gel electrophoresis.

### 16S rRNA Gene Sequencing and Quality Control of Data

The V4 hypervariable region of 16S rRNA gene was selected and amplified by the fusion primers 515F [GTGCCAGCMGCCG CGGTAA] and 806R [GGACTACHVGGGTWTCTAAT] under the melting temperature of 56°C with 30 cycles. The DNA sequencing procedure was performed on the MiSeq platform (Illumina, USA) according to the manufacturer’s manuals. All 16S rRNA gene sequencing data were submitted to the SRA database in NCBI with the accession numbers SRR4422912, SRR4422947, SRR4422914, SRR4422951, SRR4431318, SRR4431319, SRR4431321, SRR4454082, SRR4454119, and SRR4431322. Data processing and quality control were processed by the standard protocols of bioinformatics analysis. In brief, to obtain the clean sequence reads, we used custom scripts to remove the primer, low-quality, and barcode sequences. According to the report by Fu et al. (2015), we rarefied the library size to 20,000 clean reads depth. And then, FLASH (v.1.2.11) was used to assemble the paired-end clean reads into tags (Magoc and Salzberg, 2011). Unique bacterial sequences with 97% sequence similarity were clustered as operational taxonomic unit (OTU) using the QIIME software (the toolbox for Quantitative Insights Into Microbial Ecology), which uses UCLUST (an algorithm to cluster sequence reads based on similarity) to perform the clustering (Edgar, 2010). Those OTUs which had relative abundance <0.1% and were present in less than 1% of the experimental pigs were removed from further analysis. OTUs were matched to bacteria by using a primer-specific version of the GreenGenes (v13.5) reference database (DeSantis et al., 2006). A total of 234 and 243 pigs which had both phenotypes and 16S rRNA gene sequencing data were remained for the further association study between phenotypic value of fatness and relative abundance of gut microbiota in the two populations.

## Statistical Analysis

### Microbial Diversity Analysis

The α-diversity indexes of chao1, ACE, observed species, Simpson, and Shannon index were calculated by Mothur software (Schloss et al., 2009). The comparison of α-diversity indexes between *Bamaxiang* and *Erhualian* pigs was performed by Wilcoxon *t*-test. The possible correlations between the relative abundance of bacteria and the variables of environmental and host factors including pen, batch, kinship, and sex were examined by canonical correspondence analysis (CCA) using the R software with vegan package (Dixon, 2003).

### Two-Part Model for Association Analysis

To identify the gut microbes which were associated with porcine fatness, we performed the association studies with two-part model (Fu et al., 2015). The two-part model includes a binary model and a quantitative model. The binary model accounted for the effect of the presence/absence of the gut flora on porcine fatness, and the quantitative model analyzed the effect of the abundance of the microbes on porcine fatness.

To further evaluate whether the effect came from the presence/absence or the abundance of the gut microbiota or both, a combination of the binary and quantitative analysis was characterized by a meta-analysis in which the *P*-value was derived using an unweighted *Z* method. The formulas of the three models were described as below:

$$Binary\,\,\mathrm{mod}el\,:\;y=\beta_{1}b+e$$

$$Quantitative\,\;Model\,:\;y=\beta_{2}q+e$$

$$Unweighted\,\;method\,:\;Z=\overset{k}{\underset{j=1}{\Sigma }}z_{i}/\sqrt{k}\sim N\left(0,1\right);\;z_{i}=\theta^{-1}\left(P_{i}\right)$$

Where *y* refers to the trait value (backfat thickness, leaf fat weight, and abdominal fat weight) per individual after adjusting for sex and body weight, *b* is a binary feature, *q* is a quantitative feature, β_1_ and β_2_ are the estimated effects for the binary and abundance effect, and *e* represents the residuals. *Z*_i_ is the *Z*-transform test converting the one tailed *P*-values, *P*_i_ was from each of *k* independent tests into standard normal deviates. *Z* is the sum of these *Z_i_* divided by the square root of the number of tests, *k* has a standard normal distribution (Whitlock, 2005). The minimum of the *P*-values from the binary analysis, quantitative analysis, and meta-analysis was set as the final association *P*-value. We performed 1,000× permutation tests to control the false discovery rate (FDR). The FDR ≤ 0.1 was set as the significant threshold.

$$The\,\;FDR\,\;control\,:\;FDR=N_{0}/N_{1}\times 1,000\leq 0.1;$$

Where *N*_0_ is the average number of the detected significance at a certain *P* cutoff in 1,000 permutations, *N*_1_ is the number of the detected positive in the real analysis.

### Estimating the Phenotypic Variance Explained by the Gut Microbiome

To test the phenotypic variation of porcine fatness explained by gut microbiome, we conducted a 100 times cross validation. Thus, we split the data randomly into a 70% discovery set and a 30% validation set. In the discovery set, a total of *n* number of significantly associated OTUs was identified at a certain *P*-value, and the effect sizes of binary and quantitative features of each OTU (β_1_ and β_2_) were estimated before. Then, the risk of the gut microbiome on fatness traits (*r*_m_) for each animal in the validation set was calculated using an additive model:

$$r_{m}=\overset{n}{\underset{j=1}{\Sigma }}\,\left(\beta_{1}+b_{j}+\beta_{2\,j}\,q_{j}\right);$$

The phenotypic variance explained by the gut microbiome was represented as the squared correlation coefficient (*R*^2^) between the trait values corrected for sex and body weight and *r*_m_. To ensure the stability of the estimation, we repeated the cross-validation by 100 times and calculated the average value of the explained variations. Considering many microbes that may contribute a small effect but may not be confidently detected at FDR ≤ 0.1, we performed this analysis at a series of different significant *P* levels ranging from 1.0*E* – 05 to 0.1.

## Results

### Microbial Diversity in Porcine Cecum and Feces

**FIGURE 1:**
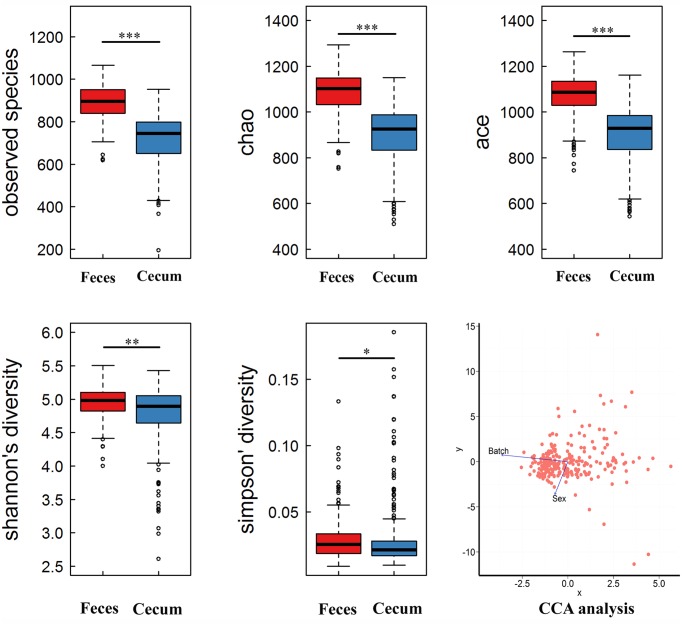
**Comparison of the α-diversity of gut microbiome between cecum and fecal samples**. The gut microbial richness was estimated by observed species, chao and ace index, the diversity was evaluated by Shannon and Simpson index. The microbial richness and diversity were showed significant difference between cecum and feces (^∗^*P* < 0.005; ^∗∗^*P* < 0.0001; ^∗∗∗^*P* < 0.00001; Wilcoxon *t*-test). Canonical correspondence analysis (CCA) showed that host gender and batch had significant effects on microbial composition (*P* < 0.05).

**FIGURE 2:**
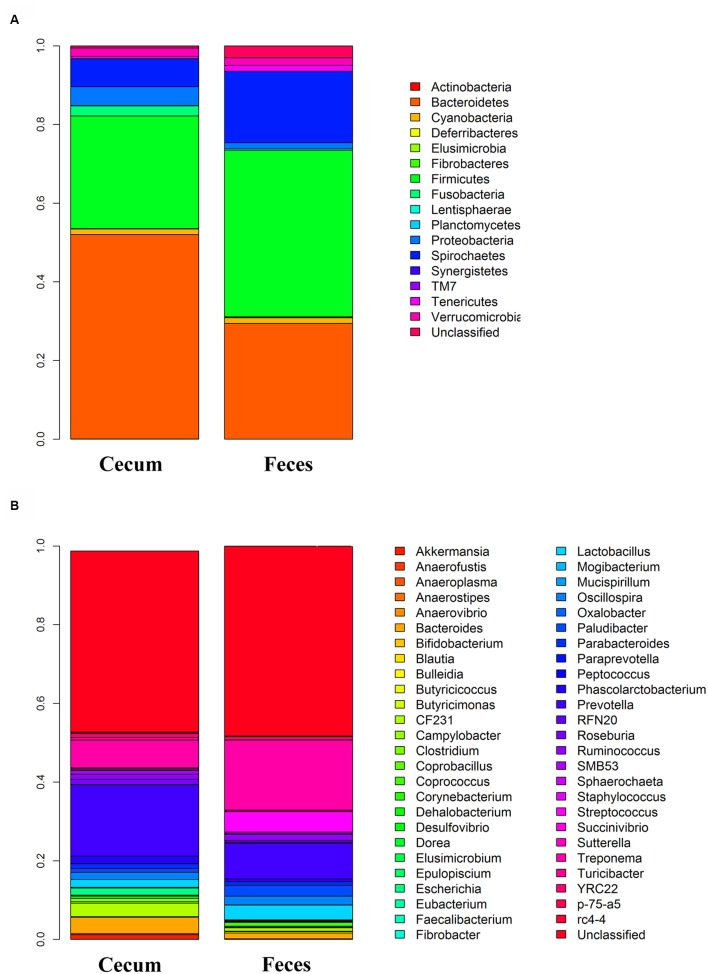
**Comparison of the relative abundance of gut microbiota between cecum and fecal samples**. **(A)** At phylum level. **(B)** At genus level.

The total numbers of sequence reads for fecal and cecum luminal samples were 8,029,976 (an average of 30,766 reads per sample) and 7,320,888 (an average of 28,597 reads per sample), respectively. We rarefied the library size to 20,000 reads per sample to reduce the effect of sequencing depth. The two pig cohorts obtained 1,660 and 2,038 OTUs. After quality control, we focused on the 610 and 524 OTUs in the *Bamaxiang* and *Erhualian* population, respectively. These OTUs occupied 99% of the total clean reads in each sample. The tags were annotated to microbial taxa. In the *Bamaxiang* population, a total of 17 phyla and 57 genera were identified. In the *Erhualian* population, the numbers of microbial phylum and genus were 18 and 99, respectively (**Table 1**). We compared the α-diversity of microbiota between cecum and feces samples using the chao1, ACE, observed species, Shannon and Simpson index, and found that all five indexes showed significant difference (*P* = 6.64*E* - 52, 1.96*E* - 50, 2.20*E* - 55, 5.76*E* - 05, and 3.90*E* - 03, respectively). The fecal samples had a significantly higher α-diversity (**Figure 1**). We further compared the phylogenetic composition of the microbial community at the phylum and genus level. The cecum luminal samples had significantly higher abundances of the phyla *Bacteroidetes* and *Proteobacteria*, and the genera *Akkermansia*, *Bacteroides*, *CF231*, *Escherichia*, and *Prevotella*. However, the fecal samples showed the higher abundances of the phyla *Firmicutes* and *Spirochaetes*, and the genera *Lactobacillus*, *Streptococcus*, and *Treponema* (*P* < 0.05) (**Figure 2**). We performed CCA analysis in the tested samples and identified the significant effects of host gender and batch on the microbial composition of both cecum and feces (*P* < 0.05).

### Identification of Gut Microbes Associated with Porcine Fatness in the Cecum and Fecal Samples

**FIGURE 3:**
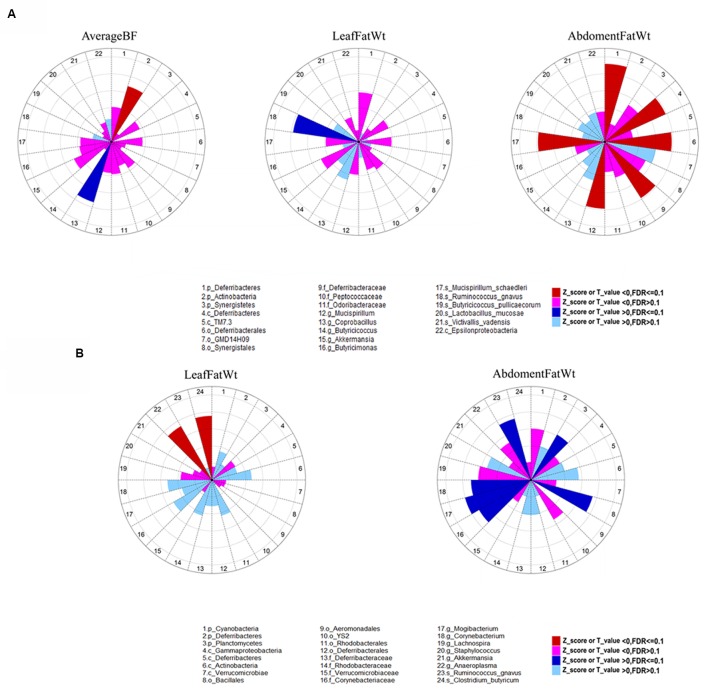
**The effect of bacterial taxonomies on abdominal fat weight, leaf fat weight, and average backfat thickness**. The effects of bacterial taxonomies on abdominal fat weight, leaf fat weight, and average backfat thickness are shown as *Z* or *T* scores in the feces **(A)** and cecum **(B),** respectively. The different color sectors indicate positive or negative associations and their significance level. Dashed circles indicate the scale of *Z* or *T*-values from 1 to 5.

The summary description of phenotypic values of backfat thickness (subcutaneous fat), leaf fat weight and abdominal fat weight is shown in **Table 2**. The phenotypic values were firstly adjusted for the effects of sex and body weight, and then the residuals were used for association analyses. In the fecal samples, we identified six OTUs (Otu363, Otu393, Otu206, Otu95, Otu1330, and Otu500) that were significantly associated with leaf fat weight at FDR ≤ 0.1. These six OTUs were annotated to unclassified *Ruminococcaceae*, *Ruminococcus gnavus*, *Lachnospiraceae*, *Firmicutes*, *Prevotella*, and *Clostridiales*, respectively. We did not identify any significant associations at OTU level for other fatness traits (Supplementary Table 1). At the taxonomic level, we identified 11 significant associations related to nine unique taxonomies for fatness traits at the significant threshold of FDR ≤ 0.1. Of the 11 associations, two were identified for AverageBF. While only one significant association was found for each of LeafFatWt, ChestBF, and WaistBF (**Figure 3A**; Supplementary Table 2). The other six associations were detected for abdomimal fat weight (AbdomenFatWt). For more details, *Actinobacteria* showed a negative association with both WaistBF and AverageBF (*P* = 6.59E-04 and 7.73*E* - 04, respectively); *Coprobacillus* was positively associated with both ChestBF and AverageBF (*P* = 1.91*E* - 04 and 2.68*E* - 04, respectively); and species *R. gnavus* was positively associated with LeafFatWt (*P* = 1.00*E* - 04). The species *Mucispirillum schaedleri* was the only member of the phylum *Deferribacteres* identified in this study. The strongly negative associations with AbdomenFatWt were identified on this microbe from phylum to species level (*P* = 1.10*E* - 04); In addition, of these 11 significant associations, eight were detected by the binary analysis (presence/absence), two were identified by the quantitative analysis (the abundance of bacteria) and one by meta-analysis.

**Table 2:** Summary description of phenotypic values of porcine fatness traits in the *Bamaxiang* and *Erhualian* population.

	*Bamaxiang* (*n* = 234)		*Erhualian* (*n* = 243)	
Fatness traits	Mean ±*SD*	Range	Mean ±*SD*	Range
ShoulderBF (cm)	4.96 ± 0.78	2.9–7.1	4.31 ± 0.87	1.8–6.3
ChestBF (cm)	4.02 ± 0.72	0.4–5.7	3.80 ± 0.82	1.5–6.1
WaistBF (cm)	2.96 ± 0.61	1.3–5.5	2.47 ± 0.69	0.7–4.6
AverageBF (cm)	3.79 ± 0.58	2.4–5.7	3.37 ± 0.72	1.2–5.3
LeafFatWt (kg)	2.07 ± 0.58	0.8–3.9	2.76 ± 0.89	0.6–5.7
AbdomenFatWt (kg)	0.72 ± 0.23	0.2–1.6	0.96 ± 0.32	0.2–1.8

*ShoulderBF, shoulder backfat thickness; ChestBF, chest backfat thickness; WaistBF, waist backfat thickness; AverageBF, average backfat thickness; LeafFatWt, leaf fat weight; AbdomenFatWt, abdominal fat weight*.

**FIGURE 4:**
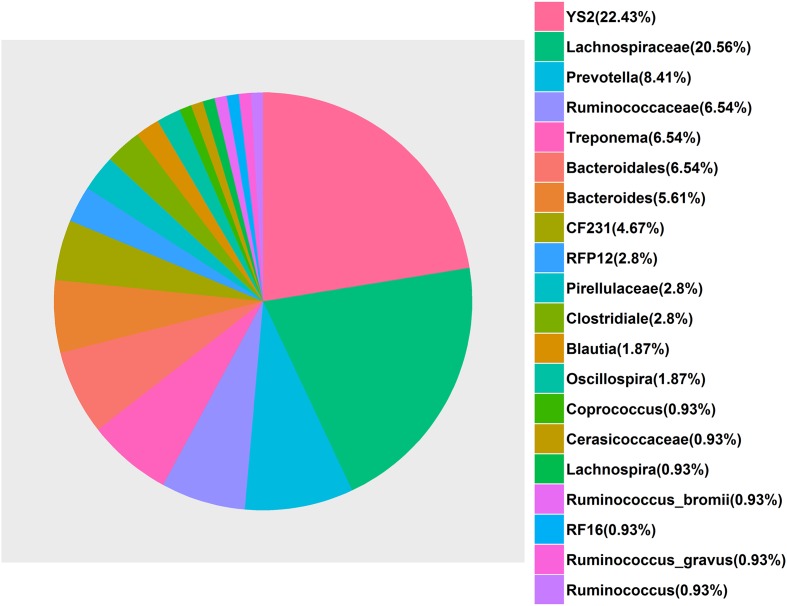
**The bacterial annotation of the 80 fatness-associated operational taxonomic units (OTUs) in the *Erhualian* population based on the Greengenes database**. The percentages were calculated with (the number of the fatness-associated OTUs annotated to a given bacterial taxonomy/80 × 100%).

With respect to the cecum luminal samples, a total of 108 significant associations for 80 unique OTUs were found at FDR ≤ 0.1, including four associations with ShoulderBF, 30 with WaistBF, 19 with the AverageBF and 55 with AbdomenFatWt. However, we did not detect any OTUs significantly associated with ChestBF and LeafFatWt. Of the 80 fatness-associated OTUs, two (Otu148 and Otu162) were shared by four traits (AbdomentFat, WaistBF, ShoulderBF, and AverageBF), three OTUs were shared by AbdomentFat, WaistBF and AverageBF, nine by WaistBF and AverageBF, and three by AbdomentFat and WaistBF. Each of the other fatness-associated OTUs was specifically associated with only one phenotype. The detailed annotation results for the fatness-associated OTUs are shown in Supplementary Table 3 and **Figure 4**. We observed that these OTUs were mainly annotated to the *YS2* (*Cyanobacteria*), *Lachnospiraceae*, *Ruminococcaceae*, *Prevotella*, *Treponema*, and *Bacteroides*. Further, the OTUs annotated to the *Ruminococcaceae* were showed positive associations with fatness traits. Those annotated to the *Prevotella*, *Treponema*, and *Bacteroides* were negatively associated with fatness traits. And both positive and negative associations were observed for the OTUs annotated to the *Lachnospiraceae*. At the taxonomic level, we identified 10 taxa that were significantly associated with fatness traits at FDR ≤ 0.1, including one for WaistBF, one for ChestBF, six for AbdomenFatWt, and two for LeafFatWt (**Figure 3B**; Supplementary Table 2). We did not identify any significant associations with ShoulderBF and AverageBF. Of the 10 fatness-associated taxa, five were detected by the binary analysis, one by the quantitative analysis and four by the meta-analysis.

*Ruminococcus gnavus* showed a positive association with both leaf fat weight in the fecal samples (*P* = 1.00*E* - 04, *Z*_score = 3.89) and abdominal fat weight in the cecum luminal samples (*P* = 1.88*E* - 04, *Z*_score = 3.73). We did not detect any other taxa that showed the significant association with fatness in both types of samples.

### Phenotypic Variance of Porcine Fatness Explained by Gut Microbiome

**FIGURE 5:**
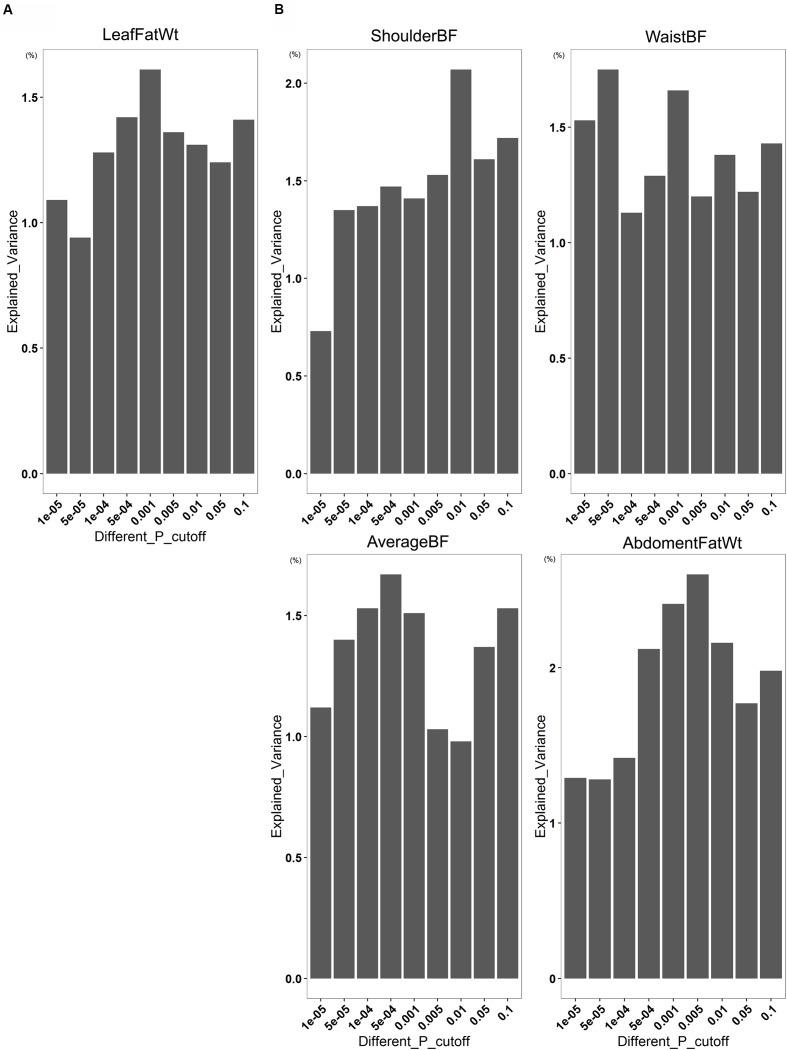
**The contribution of gut microbiome to pig fatness traits based on the associated OTUs**. The figures show the variation of fatness trait values explained by gut microbes at different significance levels in the *Bamaxiang* **(A)** and *Erhualian* **(B)** pig population.

To investigate how much degree of phenotypic variance of fatness was explained by the gut microbiome, we conducted a 100 times cross-validation analysis by splitting the data set randomly into an 70% discovery set and a 30% validation set at the OTU level. In the *Bamaxiang* pigs, we found that the OTUs identified at *P* = 1.0*E* - 05 level in the discovery set could explain 1.09% phenotypic variation of LeafFatWt in the validation set. In the *Erhualian* cohort, at *P* = 1.0*E* - 05 level, the fatness-associated OTUs can explain 0.73% phenotypic variation of ShoulderBF, 1.53% of WaistBF, 1.11% of AverageBF, and 1.29% of AbdomenFatWt. When the significance threshold of association increased to *P* = 0.1 and the risk model included more (but less significant) OTUs, the explained variance increased to 1.61% in LeafFatWt for *Bamaxiang* pigs, and 2.07% in ShoulderBF, 1.75% in WaistBF, 1.55% in AverageBF, and 2.41% in AbdomenFatWt for *Erhualian* pigs (**Figure 5**).

## Discussion

For recent years, more and more researches have concentrated upon the microbiota inhabiting the host gastrointestinal tract since gut microbiome has been reported to associate obesity and metabolic dysfunctional diseases in both humans and mice. Exploring the effect of the gut microbiome on obesity and insulin resistance has gained insight into the role of the microbiota in the development of diabetes mellitus and cardiovascular disease. Fatness is an importantly economic trait in pig production. While no such studies have been reported in pigs. To our knowledge, for the first time, we evaluated the association of the gut microbiome with fatness in swine, especially with the samples from cecum. Moreover, we adopted a novel and powerful two-part model association analysis to interpret effects of both presence/absence and relative abundance of gut microbiota on porcine fatness.

As we expected, although all experimental pigs were raised in the same farm house and fed the similar formula diet, we observed distinct phylogenetic composition of gut microbiome among samples. This should be explained by (1) different sampling sites (cecum vs. feces). Microbiota in stool are a mix mucosally associated microbes, most from the colon and lumenal microbes (Eckburg et al., 2005); (2) different genetic background between two pig cohorts.

We noticed that most of the associations were trait-specific. This heterogeneity may be caused by distinct mechanism of fat deposition for different types of adipose involving different microbes among abdominal adipose, leaf fat and subcutaneous adipose. This condition was similar to that in genetic dissection of porcine fatness traits, in which different genomic loci were identified for each fatness trait (Qiao et al., 2015). To the best of our knowledge, this study first evaluated the fatness-associated microbes in the cecum samples. Compare with the fecal samples, significantly higher number of the fatness-associated OTUs was identified in the cecum samples. As we have well known, cecum has the great diversity and complex of microbiota (Looft et al., 2014). And fermentation of dietary indigestible fiber and polysaccharides occurs at the cecum. We suggested that the samples from the cecum would be better for studying the association between microbiota and fatness than stool samples.

Interestingly, many fatness-associated microbiota identified in this study have potential functions related to metabolisms. At the taxonomic level, some of the fatness-associated bacterial taxa have been reported to participate in the process of the utilization of undigested carbohydrates from the diets or the host polysaccharide. For examples, *R. gnavus* was positively associated with fatness traits in both fecal and cecum samples. *R. gnavus* plays a pivotal role in UDCA formation in the colon, which regarded as a supplement of the bile acid (Lee et al., 2013). A recent study found that the α-galactosidase 1 (Aga1) and α-galactosidase 2 (Aga2) which are two kinds of the glycoside hydrolase (GH) family from *R. gnavus* played an indispensable role in the degradation of dietary oligosaccharides and exerted a tremendous fascination on designing of galacto-oligosaccharide (GOS) prebiotics (Cervera-Tison et al., 2012). The studies in human and rat found that *R. gnavus* was enriched in the obese rats and humans (Petriz et al., 2014; Andoh et al., 2016). Furthermore, both *R. gnavus* and *Coprobacillus* identified in this study could ferment the indigested polysaccharide into the SCFAs from the food in gastrointestinal tract, and then the SCFAs were absorbed by the host and could regulate host body energy homeostasis (Layden et al., 2013). Both *Anaerovibrio* and *Clostridium butyricum* were negatively associated with LeafFatWt. A previous study indicated that the *Anaerovibrio lipolytica* from *Anaerovibrio* can produce lipase in hydrolysis of triglyceride (Henderson, 1971). Previous studies have summarized that, as a kind of probiotics, *C. butyricum* can produce butyrate that provides the majority energy to the gut epithelial and repairs the intestinal mucosa damaged by virus (Araki et al., 2002, 2004; Zhang et al., 2011). The *M. schaedleri* was negatively associated with AbdomenFatWt in fecal samples. Interestingly, in the diet induced obesity (DIO) mice, the abundance of *Mucispirillum* was decreased (Clarke et al., 2013). *Actinobacteria* which was negatively associated with both average and waist backfat, has been recognized as the producer of many bioactive metabolites including antibacterials and antivirals for humans (Mahajan and Balachandran, 2011), and growth promoting substances for plants and animals (Atta and Ahmad, 2009).

At the OTU level, the fatness-associated OTUs were mainly annotated to YS2 (*Cyanobacteria*), *Lachnospiraceae*, *Ruminococcaceae*, *Prevotella*, *Treponema*, and *Bacteroidales* (**Figure 3**). *Lachnospiraceae* is abundant in the digestive tract of many mammals and relatively rare elsewhere. Members of this family have been linked to obesity in humans (Cho et al., 2012), mainly due to the association of many species within the group with the production of butyric acid (Duncan et al., 2002). *Ruminococcaceae* has been reported to play the role of biohydrogenation, and is capable of producing butyrate via fermenting various substrates, which may exert potential physiological functions in host health (Huws et al., 2011; Onrust et al., 2015). Kim et al. (2012) observed that *Ruminococcaceae* was enriched in mice fed high fat diet. Geurts et al. (2011) also observed a higher abundance of *Ruminococcaceae* in the *db/db* mice compared to lean mice. *Prevotella* and *Treponema* belong to xylan-degrading bacteria, which contain a set of bacterial genes for cellulose and xylan hydrolysis, and act as a host mutualistic component to help to degradate dietary fiber which could produce significantly more short-fatty acids (Warnecke et al., 2007; Flint et al., 2008). De Filippo et al. (2010) proposed that the degrading polysaccharide-rich diet allowed gut microbiota to maximize energy intake from fibers diets, but also protect them from inflammation and noninfectious colonic diseases. Besides *Prevotella* and *Treponema*, *Bacteroides* was also annotated to the fatness-associated OTUs. These microbes ferment polysaccharides to short-chain fatty acids, such as propionate by *Bacteroides* spp. from succinate pathway and acetate by *Prevotella* spp. from pyruvate via acetyl-CoA. Maslowski et al. (2009) and Smith et al. (2013) showed that the interactions of acetate and propionate with GPR43 have an important role in anti-inflammatory effects via the modulation of cT_Reg_ cell. In addition, the SCFAs can influence host homeostasis, inhibit the accumulation of fat mass development in adipose tissue and promote leptin level. In diet-induced obese mice, *Bacteroides*-*prevotella* also showed a negative correction with fat mass development and inflammation (Neyrinck et al., 2011; Ridaura et al., 2013; Ivarsson et al., 2014). In the cecum samples, *Prevotella*, *Bacteroides*, and *Treponema* were negatively associated with fatness traits.

In addition, *Blautia*, *Coprococcus*, *Ruminococcus*, and *Clostridiales* were also annotated to the fatness-associated OTUs in the cecum samples. These bacteria were identified in a phylo-functional core of gut microbiota in healthy young Chinese cohorts (Zhang J. et al., 2015). The members of *Blautia* and *Ruminococcus* have been reported to produce acetate via acetyl-CoA from pyruvate and Wood–Ljungdahl pathway by fermenting glucose and indigestible diet fiber (Miller and Wolin, 1996; Barcenilla et al., 2000; Pryde et al., 2002; Liu et al., 2008; Kovatcheva-Datchary et al., 2009; Crost et al., 2013). Perry et al. (2016) reported that the increased acetate could promote hyperphagia and increase energy storage as fat. In mice, ingestion of high fat diet was associated with the higher abundance of *Clostridiales* compared to the low fat diet regardless of propensity for obesity (de La Serre et al., 2010; Delzenne and Cani, 2011).

We estimated that gut microbiome could explain 1.55–2.73% of the variation in the six fatness-associated traits. This effect size was similar to that of most quantitative trait loci (QTL) previously reported for porcine fatness traits (Ai et al., 2012). However, in humans, gut microbiota has been implicated as a pivotal contributing factor in diet-related obesity (Zhang C. et al., 2015). This different contribution size should be due to the reason that all experimental pigs were raised in the same farm house and provided the same diet. The environmental factors, such as diets displayed less effects on gut microbiome and the subsequent fat deposition. Although the effect size of gut microbiome on fatness is small, we have established that gut microbiome should be a risk factor for porcine fatness.

A recent study reported by Ross et al. (2013) described an efficient methodology for predicting complex traits from quantitative microbiome profiles, and demonstrated that microbiome profiles can be used to predict human inflammatory bowel disease (IBD) status and BMI, and methane production in cattle with high accuracy. And based on the phylotypes of gut microbiota, Zhang et al. (2010) used partial least square discriminate analysis (PLS-DA) to predict host genotypes, diets or obesity phenotypes. In the futural study, the construction of prediction methodology based on the gut microbiome profiles for porcine fatness traits would greatly promote the pig production by reducing fat mass and improving the feed efficiency. Furthermore, the results from this study gave important cues for isolation of the causative microbes for porcine fatness that would provide basic information for regulating the gut microbiome to reduce fat deposition in pigs.

## Conclusion

We identified a number of taxa and OTUs that showed significant associations with porcine fatness traits in the cecum luminal samples and feces. The fatness-associated microbiota were mainly involved in fermenting dietary indigestible fiber and polysaccharides to produce short-fatty acids. The short-fatty acids have been reported to inhibit fat mass development and inflammation. Significantly higher number of fatness-associated OTUs were identified in the cecum suggesting that cecum luminal samples would be better used for investigation of fatness-associated microbes than stool samples. Although the effect size of gut microbiome on porcine fatness is not very large in this study, we established that gut microbiome should be a risk factor for porcine fatness. These results help us to better understand the structure and functional potential of swine gastrointestinal microbiota, and provide a new insight into the role of gut microbes in affecting the porcine fatness traits.

## Ethics Statement

All samples were collected according to the guidelines for the care and use of experimental animals established by the Ministry of Agriculture of China. Animal Care and Use Committee (IACUC) in Jiangxi Agricultural University specifically approved this study. All the experimental pigs used in this study were raised in the farm house affiliated to State Key Laboratory for Pig Genetic Improvement and Production Technology, Jiangxi Agricultural University. The pig owners consented to this study. No vulnerable populations were involved in this study.

## Author Contributions

CC conceived and designed the experiments, analyzed the data, wrote, and revised the manuscript; MH performed the experiments, analyzed the data and wrote the manuscript; SF, XH, HY, ZL, and JG performed the experiments; YZ and SK collected the samples; LH conceived and designed the experiments, and revised the manuscript.

## Conflict of Interest Statement

The authors declare that the research was conducted in the absence of any commercial or financial relationships that could be construed as a potential conflict of interest.
